# Defining the abscopal effect in non-small cell lung cancer in the era of immunotherapy and lung ablation treatment: a narrative review

**DOI:** 10.3389/fmed.2026.1804711

**Published:** 2026-04-23

**Authors:** Illaa Smesseim, Phillip N. Perez, Abraham Chachoua, Benjamin T. Cooper, Daniel H. Sterman

**Affiliations:** 1 Department of Thoracic Oncology, Netherlands Cancer Institute, Amsterdam, Netherlands; 2 Department of Pulmonary Diseases, Leiden University Medical Center, Leiden, Netherlands; 3 NYU Pulmonary Oncology Research Team (NYU PORT), Division of Pulmonary, Critical Care & Sleep Medicine, Department of Medicine, NYU Langone Health, New York, NY, United States; 4 Department of Medicine, NYU Grossman School of Medicine, New York, NY, United States; 5 Department of Radiation Oncology, NYU Langone Health, New York, NY, United States

**Keywords:** ablation, abscopal, abscopal effect, lung cancer, NSCLC, radiotherapy

## Abstract

The abscopal effect, first described in 1953, refers to the regression of distant, non-irradiated tumors following localized therapy. Historically considered rare, interest in this phenomenon has increased with the introduction of immunotherapy and local treatments for non-small cell lung cancer (NSCLC). This review summarizes the current evidence on the pathophysiology, clinical observations, and assessment of the abscopal effect in NSCLC following radiotherapy, lung ablation, and combined multimodality therapies. Preclinical and early clinical studies suggest that radiotherapy and ablative techniques such as cryoablation, microwave ablation, and pulsed electric field therapy may induce immunogenic cell death, leading to the release of tumor antigens and danger-associated molecular patterns that can activate systemic antitumor immune responses. When combined with immune checkpoint inhibitors, these local therapies may enhance immune activation, potentially improving both local and distant tumor control. However, recognition of abscopal effects remains inconsistent, largely due to limitations of conventional response assessment criteria. While iRECIST partly captures atypical response patterns, unequivocal out-of-field tumor regression is not systematically recorded in most clinical trials. The available evidence, primarily from preclinical models and early-phase studies, suggests that the true incidence of abscopal effects in NSCLC may be underrecognized. Accordingly, we propose a working definition of the abscopal effect in NSCLC: the regression (complete or partial response by iRECIST) of one or more non-irradiated lesions distant from the primary treatment site, occurring after localized therapy with or without systemic treatment, and confirmed by follow-up imaging within 4–8 weeks. Establishing standardized terminology and assessment criteria will be essential for accurately identifying and integrating potential abscopal responses in future NSCLC research and clinical practice.

## Background

The abscopal effect was first described by Mole (1), when regression of distant, non-irradiated tumors was observed following localized radiotherapy. Since then, this phenomenon has been reported in multiple cancer types, such as breast cancer, melanoma and lung carcinoma (2–4). In recent years, the treatment landscape for patients with metastatic non-small cell lung cancer (NSCLC) has changed dramatically with the introduction of immunotherapy and novel local treatment strategies such as lung ablation (5, 6). Although the abscopal effect has been described in individual case reports and some multimodality clinical trials, it seemingly remains rare. Whether this reflects its true incidence or is due to under-recognition in routine clinical practice remains unclear.

**Figure 1:**
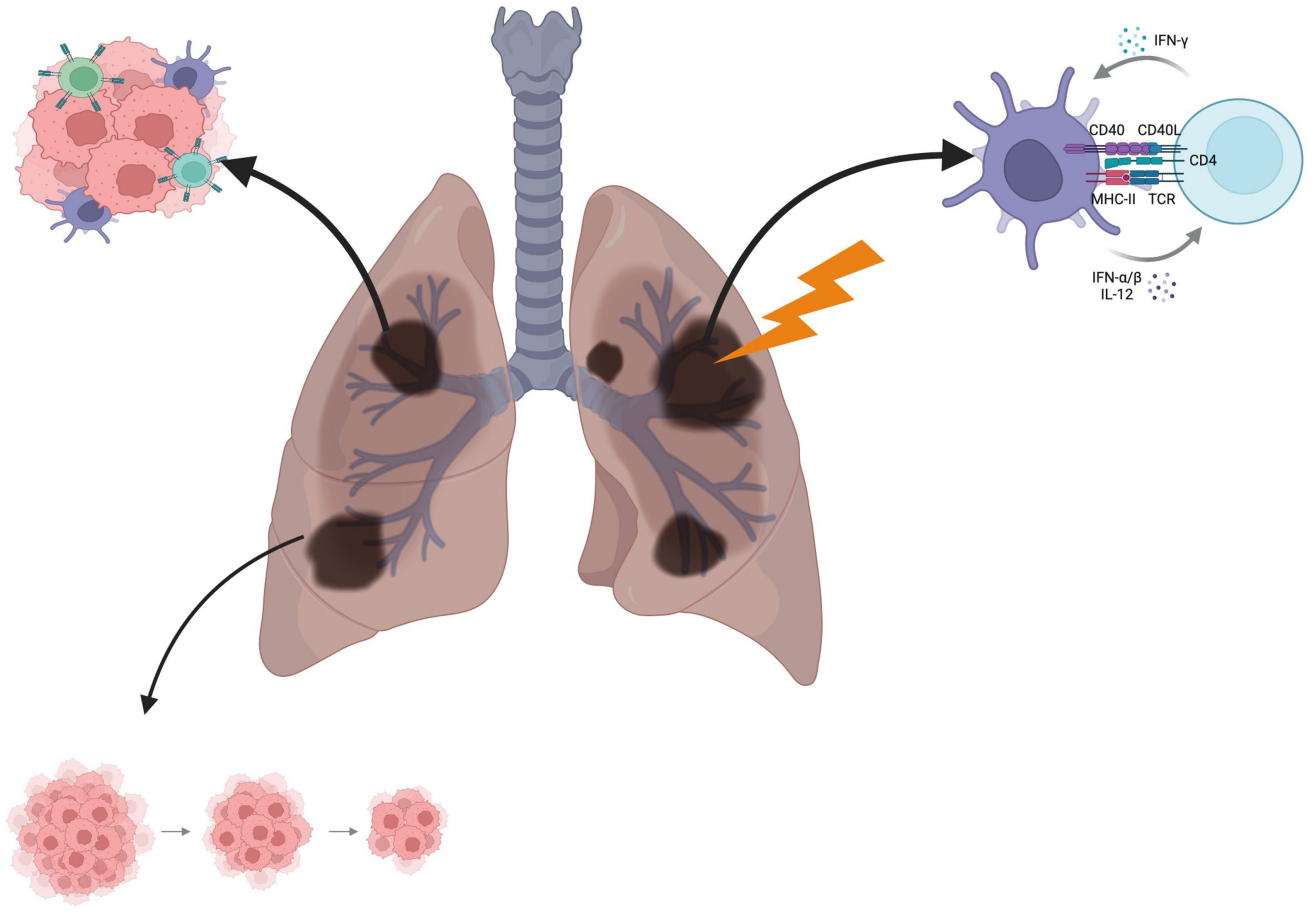
Ablative therapy induces an immune response, resulting in T-cell trafficking to metastatic sites and an abscopal effect in distant tumors. Created with BioRender.com.

Currently, treatment response in clinical trials is assessed according to RECIST version 1.1 criteria (7). Before start of treatment, target lesions are selected, with a maximum of five in total and no more than two per organ. The selection of target lesions should provide a representative reflection of the patient’s overall tumor burden. Not all lesions are eligible as target lesions however, for example, those located in areas previously treated with loco-regional therapy are considered non-measurable. Lesions not selected as targets are categorized as non-target lesions (7). Disease progression can still be determined based on these non-target lesions if there is unequivocal evidence of progression, such as a marked increase in size or number of non-target lesions. Conversely, unequivocal evidence of treatment response occurring in non-target lesions, such as abscopal effects, may not be captured within the RECIST v1.1 framework and can therefore be missed (7). To address atypical response patterns, such as pseudo-progression that is associated with immunotherapy, the iRECIST criteria were developed (8). Pseudo-progression refers to an initial increase in target size or the emergence of new lesions, followed by subsequent disease stabilization or regression, see Figure 1 (9). This can result from immune cell infiltration into the tumor microenvironment, secondary to induction of anti-tumor immune responses by systemic and/or local immunotherapy administration (e.g., checkpoint inhibition) (9).

Unlike RECIST v1.1, where such findings are immediately classified as progressive disease (PD), iRECIST introduces the category of unconfirmed progressive disease (iUPD) (7, 8). Progression must be verified on a subsequent assessment, performed at least 4 weeks later, to be designated as confirmed progressive disease (iCPD) (8).

The concept of the abscopal effect remains defined according to Mole’s (1) original description, developed in an era dominated by radiotherapy. In the context of multimodality therapies it is therefore important to clarify how abscopal effect should be assessed. Establishing standardized terminology and objective criteria is critical to ensure these responses are recognized, to allow systematic evaluation, to enable comparisons across studies, and to integrate abscopal effects into clinical decision-making.

The aim of our review is to establish a consensus definition of the abscopal effect in the treatment of non-small cell lung cancer (NSCLC), based on existing evidence from mono- and multi-modality trials, to guide future research and clinical practice.

### Pathophysiology of the abscopal effect in non-small cell lung cancer following radiotherapy

Radiotherapy modulates the tumor microenvironment by inducing both immune-activating and immunosuppressive effects (10). Ionizing radiation triggers immunogenic cell death, causing tumor cells to expose calreticulin and release danger-associated molecular patterns (DAMPs) such as HMGB1 and heat shock proteins (11). These signals, together with cytokines including TNF-α, IL-6, TGF-β, and IL-8, as well as reactive oxygen and nitrogen species, activate dendritic cells (DCs), macrophages, and natural killer (NK) cells within the tumor microenvironment (TME) (12). DAMPs engage receptors such as TLR4 on DCs, promoting MHC-I-mediated antigen presentation and DC maturation, while MHC-II upregulation facilitates CD4+ T-cell activation (10). Radiation-induced DNA damage also triggers the cGAS-STING pathway, enhancing type I interferon production, cross-presentation of tumor antigens, and priming of CD8+ cytotoxic T lymphocytes (CTLs) (13, 14) These effector T cells can traffic to distant, non-irradiated tumors, infiltrating into the TME and attacking neo-antigens on malignant cells. This results in distant tumor regression, an characteristic of the abscopal effort of radiotherapy (10, 15).

However, radiotherapy can also induce immunosuppressive mechanisms, such as accumulation of myeloid-derived suppressor cells (MDSCs) and upregulation of PD-L1 on tumor cells, which limit T-cell activity in the TME (16–18). Clinically, the abscopal effect has been documented in NSCLC, with regression of untreated metastases reported after irradiation of primary or metastatic lesions, even in patients who did not receive systemic therapy.

### Cases of abscopal effect in non-small cell lung cancer patients following radiotherapy

Several case reports have described the abscopal effect in non-small cell lung cancer (NSCLC) patients after radiotherapy. Rees et al. published one of the earliest cases, reporting regression of untreated lung metastases 20 months after radiotherapy to an oesophageal lesion in a patient with metastatic lung adenocarcinoma (19). More recently, Vilinovszki et al. (20) described an 81-year-old woman with recurrent metastatic squamous NSCLC who refused systemic therapy. Following palliative radiotherapy to the mediastinal tumor bulk, durable regression was observed both at the irradiated site and bone metastases that had not received radiotherapy. At 25 months after treatment, the patient remained in remission at all sites. Similarly, Sakaguchi et al. (21) reported abscopal regression of a vertebral metastasis after palliative irradiation of a right iliac bone lesion in a 94-year-old EGFR-mutant NSCLC patient who was unfit for chemotherapy. Other reports highlight abscopal responses following treatment of brain lesions. A case of a 60-year-old male with stage IV NSCLC (KRAS and EGFR wild type) was reported in 2009. He underwent resection of a cerebellar metastasis, followed by whole-brain radiotherapy and stereotactic radiosurgery (SRS), and subsequently received chemotherapy. The patient later developed progression of adrenal, pulmonary, hepatic, and brain lesions. The brain metastases were treated with SRS. Follow-up imaging after completion of radiotherapy showed complete resolution of extracranial disease without additional systemic therapy. The patient remained disease-free for over 5 years. Hamilton et al. (22) described complete resolution of a pleural lung mass 3 months after stereotactic radiosurgery to a solitary brain metastasis, while Takayama et al. (23) observed regression of lung and mediastinal lesions after whole-brain radiotherapy and radiotherapy of the spine in a patient with stage IV NSCLC. Kuroda et al. (24) further illustrated an abscopal effect in a 76-year-old woman with EGFR-mutant NSCLC, in whom thoracic irradiation of a hilar lymph node metastasis led to complete disappearance of pulmonary metastases on follow-up imaging. Together, these cases demonstrate that the abscopal effect in NSCLC patients can occur even after radiotherapy alone. Also, it can occur after RT to an intracerebral metastasis despite the impact of the blood–brain barrier.

### Pathophysiology of the abscopal effect in non-small cell lung cancer following lung ablation

Cryoablation induces local tumor destruction while stimulating systemic immune responses. Necrotic tumor cells release pro-inflammatory cytokines, including IL-12, IFN-γ, and TNF-α, along with tumor antigens captured by antigen-presenting cells to activate T- and B-cell responses. In partially frozen tissue, apoptosis may release immunosuppressive signals such as IL-10 and TGF-β (10, 15). Preclinical studies show that cryoablation generates tumor-specific immunity, with protection against previously ablated tumor lines and suppression of contralateral tumor growth, consistent with an abscopal effect. Mechanistically, cryoablation activates IFN-γ, IL-2/STAT5, IL-6/JAK/STAT3, and type I interferon pathways, while single-cell transcriptomics reveal enhanced antigen presentation and a transitional IFN-stimulated T-cell state that prolongs CD8+ effector activity. STING-TBK1 signaling contributes to systemic responses of cryoablation, and blockade of type I interferons reduces these effects (16). Clinically, cryoablation increases circulating CD8+ T cells, elevates CD8+/regulatory T cell (Treg) ratios, reduces FoxP3+ Tregs, and remodels the tumor microenvironment to favor immune infiltration and antiviral signaling (16, 17).

Microwave ablation (MWA) of pulmonary malignancies induces measurable immunogenic changes, characterized by an increase in cytotoxic CD8+ T cells and a decrease in Treg cells While the increase in CD8+ Tcell proportion and decrease in Treg population is widely shown, the effect on helper Tcell subtypes and associated cytokines is more unclear. A preference for the induction of the Th1 phenotype of helper T cells may be present, with one study showing a significant elevation in the Th1-associated cytokines IL-2, IFN-γ, TNF-α, and IL-12p70, while the Th2-associated cytokines IL-4 and IL-10 remained unchanged (25). Another study demonstrated a decrease in IL-2 in 59.1% of patients a month after receiving percutaneous MWA, with this being a proposed mechanism for the downregulation of Tregs (26). More studies investigating the effect of IL-2 are needed for a definitive answer.

Pulsed electric field (PEF) therapy similarly promotes immune activation after tumor cell death. PEF induces the release of DAMPs such as HMGB1, stimulating dendritic cells and tumor-specific CD8+ T cells (19–21). In addition, PEFincreases APC activity, reduces Tregs and M2 macrophages, and modulates immune signaling pathways, including IL-6, JAK–STAT, and Th17/IL-17, with early activation followed by later downregulation (22). Importantly, PEF preserves the extracellular matrix and local vasculature, maintaining tumor antigen integrity and facilitating immune cell infiltration, thereby enhancing both innate and adaptive anti-tumor responses compared to heat-based ablation (23).

### Cases of abscopal effect in non-small cell lung cancer patients following ablation

The evidence for an abscopal effect following tumor ablation in metastatic NSCLC is scarce. To date, the literature mainly consists of isolated case reports describing regression of distant untreated lesions after local ablative procedures. Shao et al. reported a 69-year-old male with advanced squamous NSCLC initially treated with four different lines of systemic therapy (27). Subsequently, oligo-progression was observed with enlargement of the primary lung tumor and mediastinal 4R/7 lymph nodes. Due to severe COPD, the patient could not tolerate radiotherapy to both sites, and CT-guided microwave ablation was performed on the primary tumor. Follow-up imaging demonstrated gradual absorption of the ablated lung lesion and concurrent shrinkage of the untreated mediastinal lymph nodes, consistent with an abscopal effect. An abscopal effect of microwave ablation has been reported in lung metastases from various tumor types, including endometrial and colorectal carcinomas, although some evidence derives from preclinical models (28, 29). While these observations raise the possibility that tumor ablation may trigger systemic immune activation through antigen release and inflammatory signaling, the current level of evidence remains limited. In contrast to radiotherapy-based strategies to induce an abscopal effect, which have been investigated in several clinical studies, the immunomodulatory effects of ablative techniques have not been well defined. Their potential role in promoting systemic antitumor immune responses requires further investigation.

### Pathophysiology of the abscopal effect in non-small cell lung cancer following radiotherapy and immunotherapy

An abscopal effect has been observed in multiple preclinical models combining radiotherapy with immune checkpoint blockade (30–37). Wei et al. (38) investigated antitumor activity at both local and distant tumor sites and found that the efficacy of the abscopal response was influenced by the timing of αPD-1 antibody delivery in relation to radiotherapy. When PD-1 blockade was applied following local irradiation, it promoted the expansion of polyfunctional CD8^+^ T cells within the tumor, reduced the population of dysfunctional CD8^+^ T cells, facilitated the reprogramming of CD8^+^ T cells, and triggered strong abscopal effects (38). Huang et al. (39) investigated the immunostimulatory impact of stereotactic body radiation therapy (SBRT) followed by pembrolizumab in patients with metastatic non-small cell lung cancer. Notably, patients with immunologically “cold” tumors achieved improved progression-free survival when SBRT preceded immunotherapy. Expression of IFN-γ, IFN-α, and antigen processing and presentation gene sets was significantly higher in nonirradiated tumor sites after SBRT. Moreover, a significant on-therapy expansion of both new and pre-existing T cell clones was observed in the non-irradiated tumor (*abscopal*) as well as the blood (*systemic*) (39).

### Prospective trials combining radiotherapy and immunotherapy in non-small cell lung cancer patients

Numerous prospective clinical trials have investigated the combination of radiotherapy and immunotherapy in patients with NSCLC (see Table 1). These studies differ in treatment combinations and primary endpoints, reflecting the heterogeneity of study objectives. Endpoints have included progression-free survival, overall survival, time to metastasis, local and regional recurrence, safety, as well as measures of systemic immune response, such as the abscopal effect. Methods for assessing tumor response have also varied. While several trials applied RECIST v1.1, others used iRECIST to capture atypical response patterns, including pseudo-progression. Moreover, two studies included out-of-field regression as an endpoint (40, 41). This variability in response assessment underscores the challenges of standardizing outcome measures in trials combining radiotherapy with immunotherapy and highlights the importance of selecting appropriate criteria to capture both local treatment response and abscopal effects.

**Table 1:** Prospective trials combining radiotherapy and immunotherapy in non-small cell lung cancer patients.

Trial	Study design	*N*	Treatment combi	Primary outcome	Definition
I-SABR (44)	Randomized phase II trial	156	Nivolumab + SABR	EFS	Local recurrence (in-field regrowth or new disease anywhere in the same lobe), regional recurrence (any intrathoracic lymph node), distant metastasis (all extrathoracic areas, as well as lung disease in any separate lobe), second primary lung cancer (SPLC)
NCT03223155 (45)	Randomized phase I trial	37	Nivolumab + ipilimumab + SBRT	PFS, OS	Clinical evaluation of patients was performed during radiotherapy and with every immunotherapy treatment. Radiographic evaluation was performed using CT scans every 12 weeks, or earlier if clinically warranted. A modified version of RECIST version 1.1 was used to assess overall response, allowing for the inclusion of both irradiated and nonirradiated metastases as target and nontarget metastases.
ASTEROID (46) (ongoing trial)	Randomized phase II trial	47	Durvalumab + SBRT	TTP	N/A
PACIFIC (42)	Randomized phase III trial	713	Durvalumab + CRT	PFS, OS	RECIST v1.1
LUN14-179 (47)	Single arm phase II trial	93	Pembrolizumab + CRT	Time to metastastic disease	N/A
DETERRED (48)	Phase II trial	40	Atezolizumab + CRT	Safety and tolerability	N/A
DOLPHIN (49)	Single arm phase trial	74	Durvalumab + Radiotherapy	PFS	RECIST v.1.1, no adjustments for abscopal effect
PACIFIC-6 (50)	Single arm phase II trial	117	Durvalumab + CRT	Toxicity	RECIST v.1.1, no adjustments for abscopal effect
KEYNOTE-001 (51)	Phase I trial	98	Pembrolizumab + RT	PFS, OS	iRECIST 4.0
PACIFIC (52)	Phase III trial	713	Durvalumab + CRT	PFS, OS	RECIST v.1.1, no adjustments for abscopal effect
SICI (53)	Phase I trial	15	Durvalumab + Tremelimumab + SBRT	Safety	RECIST v.1.1, no adjustments for abscopal effect
SWORD trial (40)	Phase II trial	55	sintilimab (anti-PD1 antibody), stereotactic body radiotherapy (SBRT) and granulocyte-macrophage colony-stimulating factor (GM-CSF)	ORR, out-of-field response rate (ASR), PFS, OS, adverse events	RECIST 1.1, abscopal effect was a secondary endpoint
Pembro-RT (43)	Prospective randomized phase I/II	148	Pembrolizumab + RT	ARR, ACR, PFS, OS	RECIST, version 1.1, by an independent reviewer. The irradiated lesion was excluded from RECIST measurements. Pseudo-progression was not scored as progressive disease for the primary endpoint.
Schoenfeld et al. (54)	Randomized phase II trial	78	Durvalumab plus tremelimumab alone or in combination with low-dose or hypofractionated radiotherapy	ORR	RECIST version 1.1, excluding the irradiated lesion. Local control within irradiated fields and abscopal response rates (data not uniformly collected and therefore not reported).
Welsh et al. (41)	Prospective randomized phase I/II trial	100	SBRT and pembrolizumab	Toxicity, best out-of-field lesion response	Immune-related response criteria. Briefly, complete response (irCR) was defined as complete elimination of all tumors, partial response (irPR) was defined as at least a 50% reduction in total tumor burden and progressive disease (irPD) was defined as *a* > 30% increase in tumor burden. All other cases were classified as stable disease (irSD). Out-of-field response referred to irCR or irPR. Progression was determined after consecutive imaging demonstrated an increase in tumor burden but was backdated to the initial time of progression.
Mattes et al. (55)	Prospective phase I trial	35	SBRT and immunotherapy (with/without chemotherapy)	Adverse events, ORR	iRECIST
Formenti et al. (56)	Prospective phase II trial	39	RT + ipilimumab	immunity-mediated tumor response outside the radiation field (abscopal effect)	iRECIST and RECIST v1.1.
Van Limbergen et al. (57)	Prospective phase I trial	6	SBRT + L19-Interleukin 2	Toxicity, PSF, OS	RECIST v1.1

EFS, event-free survival; PFS, progression free survival; ORR, objective response rate; OS, overall survival; ARR, abscopal response rate; ACR, abscopal control rate; SBRT, stereotactic body radiation therapy; RECIST, response evaluation criteria in solid tumors; RT, radiotherapy; CRT, chemoradiotherapy.

## Discussion

In our review, we reviewed evidence on the abscopal effect in non-small cell lung cancer treatment with radiotherapy, ablation, and combinations with immunotherapy. Our review highlights the variability in study design, treatment modalities, study endpoints, and response assessment methods, as well as the inconsistent consideration of out-of-field tumor regression as a marker of systemic anti-tumor activity.

The abscopal effect, which was once considered a rare phenomenon, has gained renewed relevance in the era of multimodality therapy and immune checkpoint inhibition.

Radiotherapy-immunotherapy combination trials summarized in Table 1 also provide important insights into the mechanisms underlying the abscopal effect. Although most studies were not specifically designed to evaluate out-of-field tumor regression, several trials suggest that local irradiation may enhance systemic antitumor immune responses when combined with immune checkpoint blockade. Radiotherapy can induce immunogenic cell death and promote the release of tumor-associated antigens, which facilitates antigen presentation and activation of cytotoxic T cells (13, 14). In combination with checkpoint inhibitors, this process may amplify systemic immune responses and increase the likelihood of regression of distant, non-irradiated lesions. Some trials have attempted to capture these systemic effects (40, 42, 43). For example, the Pembro-RT trial excluded irradiated lesions from RECIST measurements to better evaluate systemic tumor responses (43), while the SWORD trial included out-of-field response rate as a secondary endpoint (40). In contrast, larger trials such as the PACIFIC study primarily focused on survival outcomes and applied conventional RECIST criteria, which may underestimate the occurrence of abscopal responses (42). Collectively, these studies highlight both the potential of radiotherapy to enhance systemic immune activation and the methodological challenges in consistently identifying abscopal effects in clinical trials.

Critically, the ability to detect and quantify abscopal responses depends on the methodology used to assess tumor regression. Conventional RECIST v1.1 criteria often fail to capture all out-of-field tumor regression, leading to under-recognition of systemic effects. The iRECIST partially addresses these limitations, yet not all immunotherapy trials include these criteria when assessing tumor response. And unequivocal out-field-regression is not included as a criterion.

A key strength of our study is the comprehensive evaluation of the abscopal effect in NSCLC patients across multiple treatment modalities, including radiotherapy, ablation, and immunotherapy as a combination therapy with the other modalities. In our review, we also integrated evidence from both preclinical and clinical studies. We critically assessed the evidence by highlighting differences in study design, endpoints, and response assessment methods. This provides an understanding of how abscopal effects have been captured to date and may explain why the abscopal effect has been reported so sporadically.

Several limitations should be acknowledged. Most evidence originates from small case reports or early-phase trials, limiting the generalizability of our conclusions. Heterogeneity in treatment regimens, imaging schedules, and response criteria across studies complicates direct comparisons. Furthermore, the lack of standardized definitions and consistent reporting of the abscopal effect constrains our ability to estimate its true frequency and clinical impact in NSCLC. Based on our review, we propose an adapted definition of the abscopal effect. To implement this, it is essential that tumor response measurement criteria be expanded to include the regression of out-of-field lesions. Accordingly, we define the abscopal effect in NSCLC as follows: the regression (complete response or partial response according to the iRECIST criteria), of one or more non-irradiated tumor lesions at a distance from the primary treatment site, occurring after localized therapy, with or without concurrent systemic therapy, and confirmed by follow-up imaging (4–8 weeks). We encourage additional reports and comments from the medical community as we aim to hone this definition further. This is vital to the development of further immune-activating local therapies for advanced-stage non-small cell lung cancer.
